# Septic coronary embolism presenting as anterior STEMI: a stentless PCI strategy guided by clinical inconsistency and pathological confirmation—case report

**DOI:** 10.1093/ehjcr/ytag168

**Published:** 2026-03-10

**Authors:** Noriaki Iwahashi, Reiko Tanaka, Satoshi Fujii, Keiji Uchida, Kiyoshi Hibi

**Affiliations:** Division of Cardiology, Yokohama City University Medical Center, 4-57 Urafune-cho, Minami-ku, Yokohama 232-0024, Japan; Division of Cardiology, Yokohama City University Medical Center, 4-57 Urafune-cho, Minami-ku, Yokohama 232-0024, Japan; Department of Molecular Pathology, Yokohama City University Graduate School of Medicine, 3-11 Fukuura, Kanazawa-ku, Yokohama 236-0006, Japan; Cardiovascular Center, Yokohama City University Medical Center, 4-57 Urafune-cho, Minami-ku, Yokohama 232-0024, Japan; Department of Cardiology, Yokohama City University Graduate School of Medicine, 3-11 Fukuura, Kanazawa-ku, Yokohama 236-0006, Japan

**Keywords:** Case report, Septic coronary embolism, Infective endocarditis, Acute myocardial infarction, Intravascular ultrasound, Aortic regurgitation

## Abstract

**Background:**

Septic coronary embolism is an uncommon cause of acute myocardial infarction and may mimic atherosclerotic occlusion. Early distinction between these mechanisms is essential because routine stent implantation may be hazardous in bacteremic conditions.

**Case summary:**

A 48-year-old man presented with an anterior ST-segment elevation myocardial infarction with high fever. Coronary angiography revealed abrupt mid–left anterior descending artery occlusion with a smooth vessel contour (*Figure 1*). Intravascular ultrasound showed a large intraluminal thrombus without underlying plaque (*Figure 2*). Because these findings were inconsistent with plaque rupture, stent implantation was intentionally avoided. Pathological analysis of the aspirated thrombus demonstrated bacterial colonies (*Figure 3*), confirming septic coronary embolism due to methicillin-sensitive *Staphylococcus aureus* infective endocarditis. The patient subsequently developed acute severe aortic regurgitation secondary to left coronary cusp perforation on transesophageal echocardiography (*Figures 4* and *5*) and underwent urgent aortic valve replacement.

Learning points

- Coronary embolism should be suspected when angiographic and intravascular imaging findings are inconsistent with atherosclerotic occlusion in acute myocardial infarction.
- Pathological examination of aspirated coronary thrombus can provide definitive etiological confirmation of septic embolism.

## Introduction

Septic coronary embolism is a rare cause of acute myocardial infarction and may closely mimic atherosclerotic occlusion on angiography. Distinguishing these mechanisms during primary percutaneous coronary intervention is challenging but essential, as stent implantation in the setting of bacteremia carries substantial risks, including device infection or mycotic complications. We report a case of septic coronary embolism initially presenting as an anterior ST-segment elevation myocardial infarction, in which intravascular imaging and thrombus pathology were instrumental in reaching the correct diagnosis and guiding appropriate management. Septic coronary embolism has been reported as a rare but important cause of acute myocardial infarction.^1–3^

## Summary figure

**Figure:**
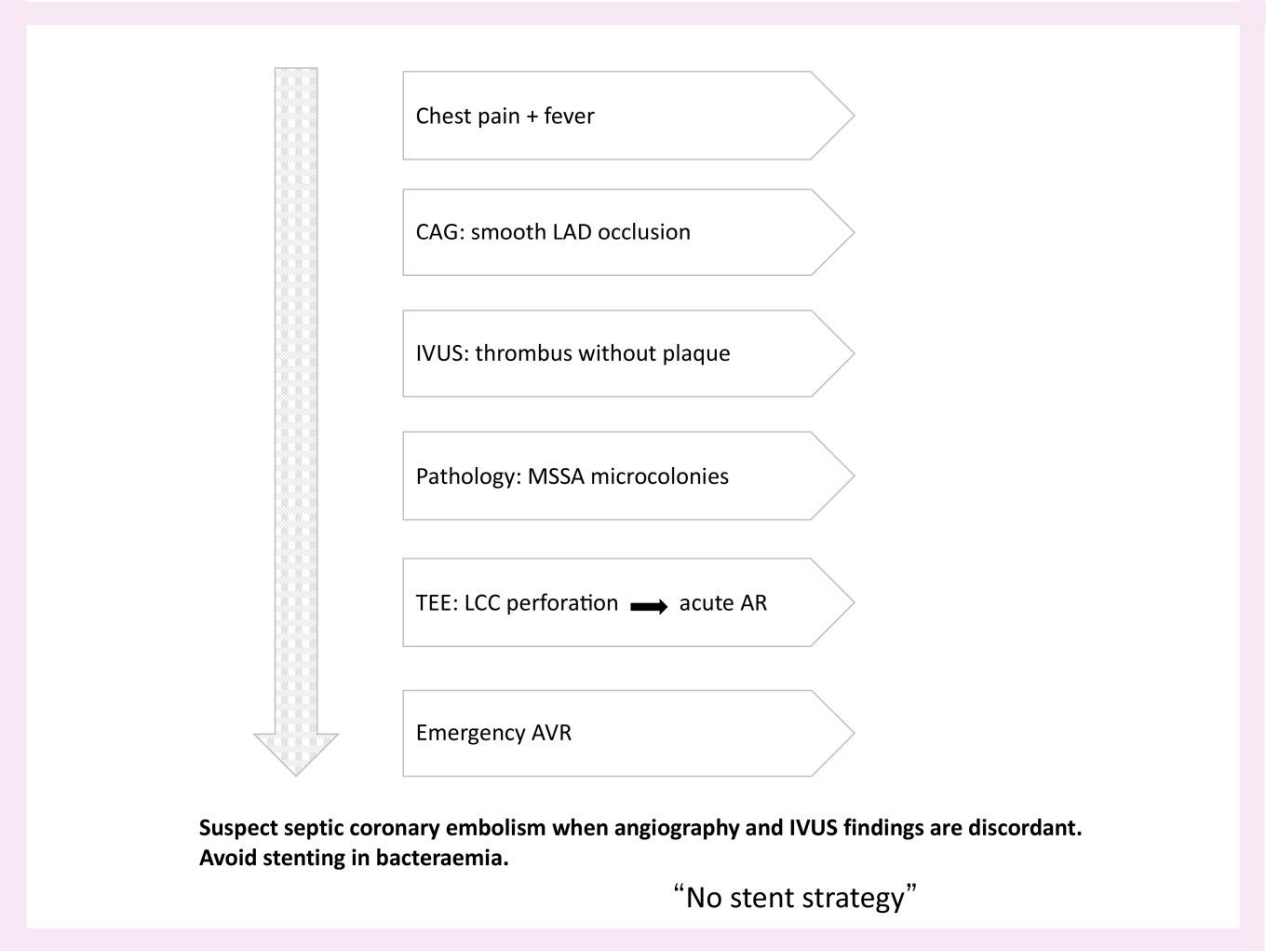


## Case presentation

**Figure 1:**
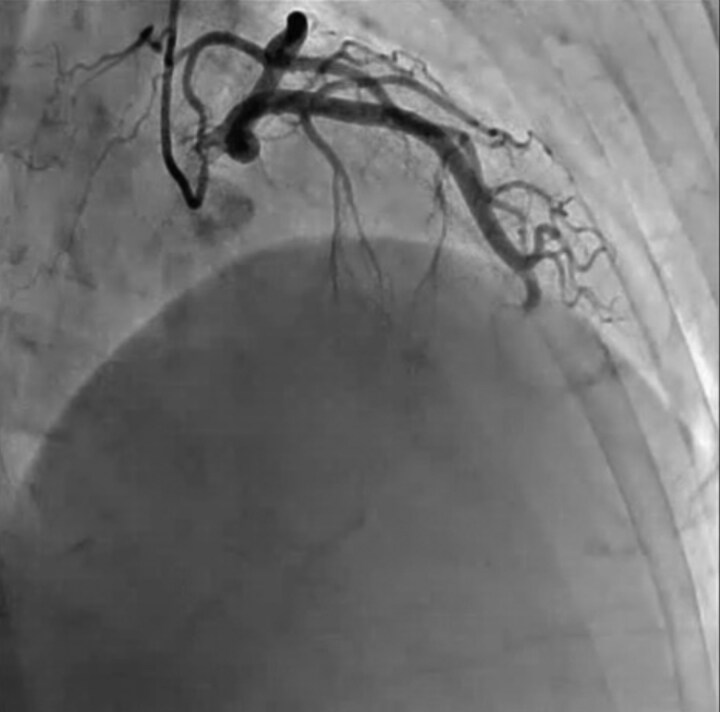
Coronary angiography. Abrupt occlusion of the mid–left anterior descending artery with a smooth, tapering contour and no angiographic evidence of underlying atherosclerotic plaque, raising suspicion of a non-atherosclerotic mechanism.

A 48-year-old previously healthy man presented with sudden-onset chest pain and persistent fever. Electrocardiography showed anterior ST-segment elevation, prompting emergent coronary angiography. Imaging demonstrated abrupt occlusion of the mid–left anterior descending artery with a smooth, tapering contour and no angiographic signs of plaque rupture (*Figure 1*).

**Figure 2:**
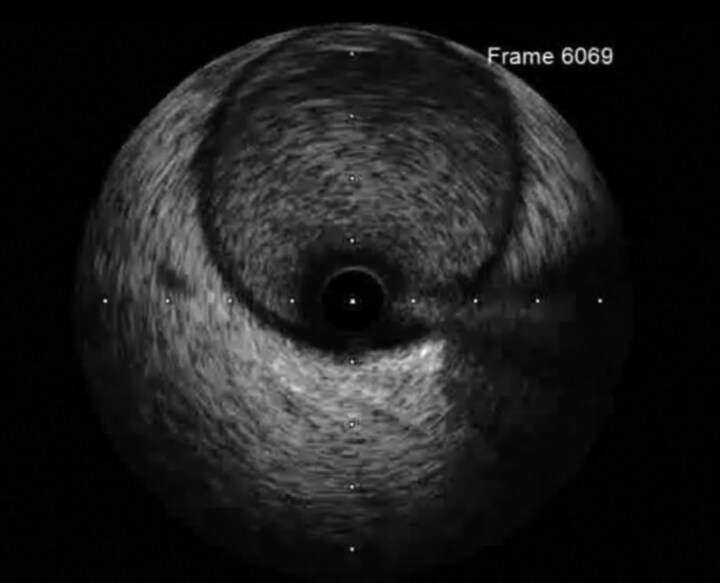
Intravascular ultrasound. A large, homogeneous intraluminal thrombus without calcification or atherosclerotic plaque. The vessel wall appears smooth and intact, inconsistent with plaque rupture or erosion.

Intravascular ultrasound revealed a large, homogeneous thrombus occupying the lumen without evidence of plaque rupture, calcification, or atherosclerotic burden (*Figure 2*). The discrepancy between the patient’s angiographic morphology and typical features of atherosclerotic occlusion raised suspicion of a non-atherosclerotic mechanism. Consequently, stent implantation was avoided, and thrombus aspiration was performed.

**Figure 3:**
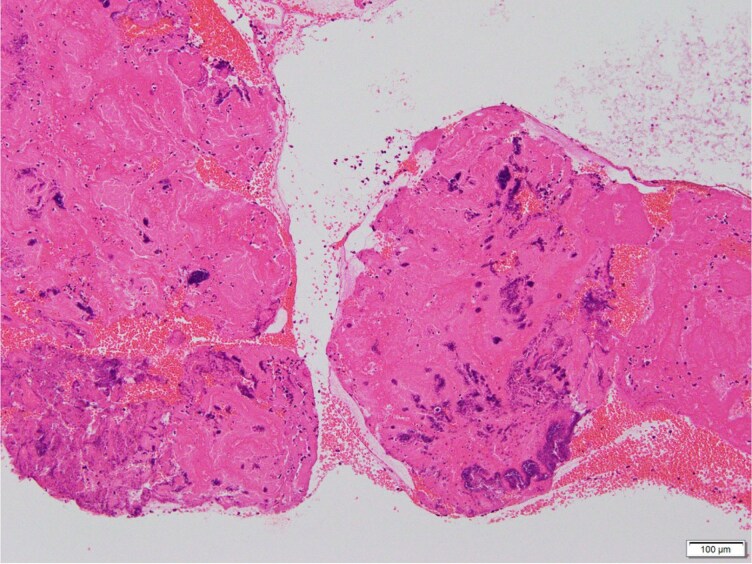
Pathological examination of the aspirated thrombus. Hematoxylin–eosin staining demonstrating basophilic (purple) microcolonies arranged in grape-like clusters within a fibrin-rich thrombus, consistent with Gram-positive cocci such as methicillin-sensitive Staphylococcus aureus.

Pathological examination of the aspirated thrombus demonstrated basophilic, grape-like microcolonies of bacteria embedded within fibrin (*Figure 3*), establishing the diagnosis of septic coronary embolism. Methicillin-sensitive *Staphylococcus aureus* was isolated from all blood culture samples obtained both during and after the catheterisation.

**Figure 4:**
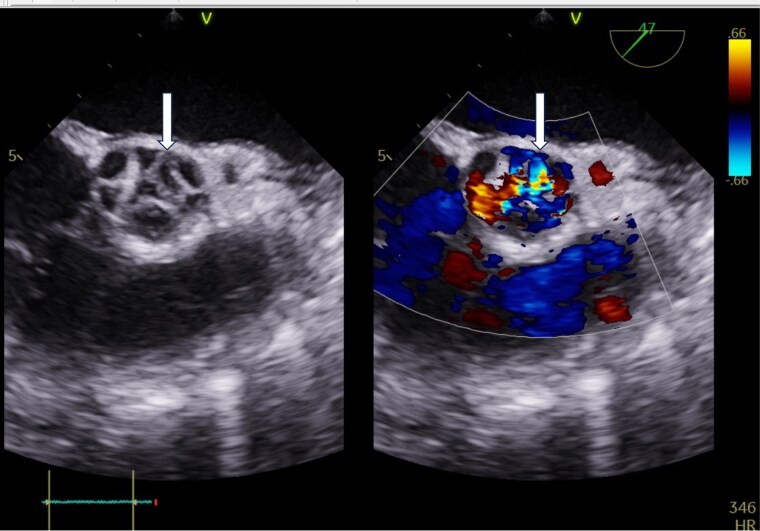
Two-dimensional transesophageal echocardiography. (*A*) Mid-oesophagal long-axis view showing perforation of the left coronary cusp without colour Doppler, demonstrating the anatomical defect caused by infective endocarditis. (*B*) Colour Doppler imaging demonstrates a simultaneous eccentric aortic regurgitation jet originating from the perforation, indicating acute valve failure.

**Figure 5:**
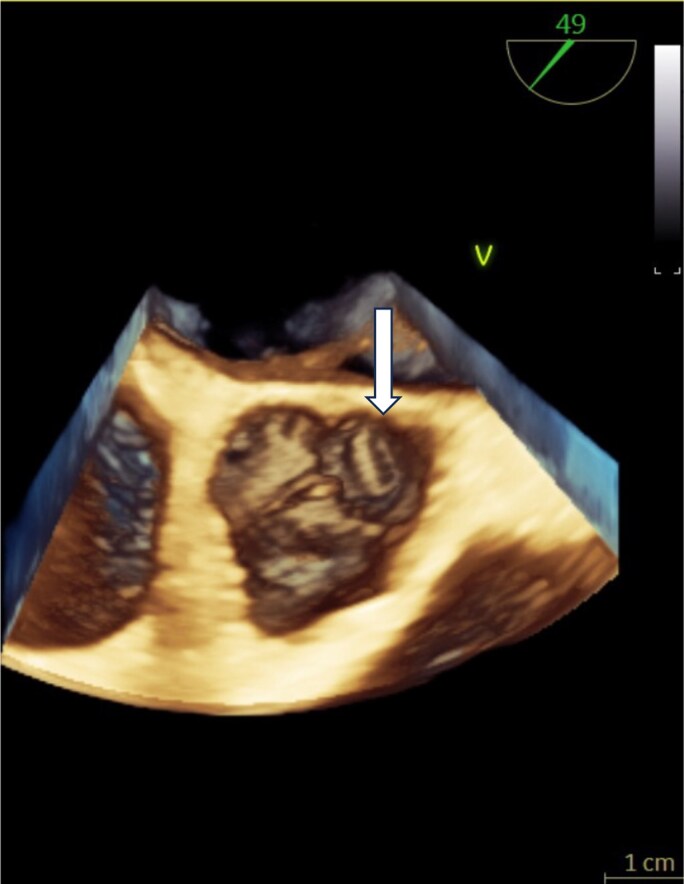
Three-dimensional transesophageal echocardiography. En-face 3D view clearly delineating perforation of the left coronary cusp, providing structural confirmation of the anatomical defect responsible for acute severe aortic regurgitation.

Transesophageal echocardiography revealed a perforation of the left coronary cusp with a simultaneous eccentric aortic regurgitation jet, confirming acute valve destruction due to infective endocarditis (*Figure 4*). Three-dimensional transesophageal echocardiography further delineated the perforation from an en face perspective, providing structural confirmation of the anatomical defect (*Figure 5*).

## Discussion

Septic coronary embolism is a rare aetiology of acute myocardial infarction, often presenting with angiographic features that overlap with conventional thrombotic occlusion. The smooth tapering morphology of the coronary obstruction (*Figure 1*) and the absence of an atherosclerotic substrate on intravascular ultrasound (*Figure 2*) were key clues indicating an embolic mechanism rather than plaque rupture.

Avoiding stent implantation was an important decision in this case. Introducing intravascular prosthetic material during active bacteremia may lead to stent infection, peri-stent abscess, or mycotic aneurysm, all associated with high morbidity and mortality. The patient’s rapid progression to acute severe aortic regurgitation—confirmed by transesophageal echocardiography showing cusp perforation (*Figures 4* and *5*)—further emphasized the severity of disseminated infection.

Definitive etiologic diagnosis was achieved only through pathological analysis of the aspirated thrombus (*Figure 3*), which demonstrated bacterial colonies. This is rarely accomplished in clinical practice but provides critical information that guides antimicrobial therapy and the need for surgical intervention.

This case underscores the importance of correlating clinical features and intravascular imaging in atypical acute coronary syndromes. In the presence of fever, systemic inflammation, and imaging inconsistencies, septic coronary embolism should be strongly considered, and a stentless strategy may be preferable. These findings are consistent with previous reports and current guideline-based understanding of infective endocarditis and its complications.^4,5^

## Conclusions

Septic coronary embolism must be recognized early when angiographic and intravascular imaging findings are discordant with typical plaque rupture. A stentless strategy supported by thrombus pathology is essential for appropriate antimicrobial and surgical management.

## Data Availability

The data underlying this article are available in the article and its online Supplementary material.

## Lead author biography

Noriaki Iwahashi, MD, PhD, is a staff cardiologist and division chief at Yokohama City University Medical Center, Japan. He specializes in cardiac imaging, particularly advanced echocardiography, and coronary interventional procedures. His clinical interests include the diagnostic integration of multimodality imaging in acute cardiovascular care and the optimisation of decision-making in complex coronary and structural heart disease. He is also actively involved in clinical research focusing on infective endocarditis, myocardial dysfunction, and hemodynamic assessment, and has contributed to the development of imaging-guided strategies in cardiovascular medicine.

## Notes

**Consent:** Written informed consent was obtained from the patient for publication of this case report.
